# Post-mortem diagnosis of Pompe disease by exome sequencing in a Moroccan family: a case report

**DOI:** 10.1186/s13256-018-1855-0

**Published:** 2018-10-29

**Authors:** Najlae Adadi, Maryem Sahli, Grégory Egéa, Ilham Ratbi, Mohamed Taoudi, Layla Zniber, Wafaa Jdioui, Said El Mouatassim, Abdelaziz Sefiani

**Affiliations:** 1 Centre de Génomique Humaine, Faculté de Médecine et Pharmacie, Mohammed V University, Rabat, Morocco; 2 Department of Medical Genetics, National Institute of Health, BP 769 Agdal, 10090 Rabat, Morocco; 3 Département de Génétique Moléculaire, Laboratoire Biomnis, Lyon, France; 4 Centre de Cardiologie, Rabat, Morocco; 5 Appolonbioteck, Brignais, France

**Keywords:** Post-mortem diagnosis, Pompe disease, *GAA* gene, Moroccan family

## Abstract

**Background:**

Pompe disease is an autosomal recessive lysosomal storage disorder characterized by progressive myopathy with proximal muscle weakness, respiratory muscle dysfunction, and cardiomyopathy. Its prevalence ranges between 1/9000 and 1/40,000. It is caused by compound heterozygous or homozygous mutations in the *GAA* gene, which encodes for the lysosomal enzyme alpha-glucosidase, required for the degrading of lysosomal glycogen.

**Case presentation:**

In this study, we report the case of a Moroccan consanguineous family with hypertrophic cardiomyopathy and sudden cardiac deaths at an early age; our patient was a 7-month-old Moroccan girl. Whole exome sequencing identified the deleterious homozygous mutation c.236_246delCCACACAGTGC (p.Pro79ArgfsX13) of *GAA* gene leading to a post-mortem diagnosis of Pompe disease.

**Conclusion:**

The identification of the genetic substrate in our patient, the daughter, confirmed the clinical diagnosis of Pompe disease and allowed us to provide appropriate genetic counseling to the family for future pregnancies.

## Background

Pompe disease, also known as glycogen storage disease (GSD) and as lysosomal storage disease, is an autosomal recessive disorder with an estimated incidence of between 1/9000 and 1/40,000 [1–3]. It is caused by compound heterozygous or homozygous mutations in the *GAA* gene, which encodes the lysosomal enzyme alpha-glucosidase (α-1,4-glucosidase) that is required for the degrading of lysosomal glycogen. Deficiency of α-1,4-glucosidase causes generalized accumulation of lysosomal glycogen in various tissues especially in the heart, skeletal system, and nervous system [4]. The clinical presentation varies widely with respect to age of onset, organ involvement, severity, and rate of progression. Infantile-onset Pompe disease presents in the first 2 months of life with hypotonia, feeding difficulties, failure to thrive, respiratory distress, hypertrophic cardiomyopathy (HCM), and hearing loss. Without treatment by enzyme replacement therapy (ERT), death commonly occurs in the first year of life from progressive left ventricular outflow obstruction [4, 5]. The late-onset form (that is, childhood, juvenile, and adult-onset) is characterized mainly by progressive muscle weakness, swallowing difficulties, and respiratory insufficiency at a slower rate. Early initiation of ERT may reduce cardiac mass and improve the ejection fraction [6].

Here we present a family with HCM and sudden cardiac deaths in multiple siblings in whom genetic testing using whole exome sequencing (WES) established a post-mortem diagnosis of Pompe disease.

## Case presentation

### Patients

**Fig. 1:**
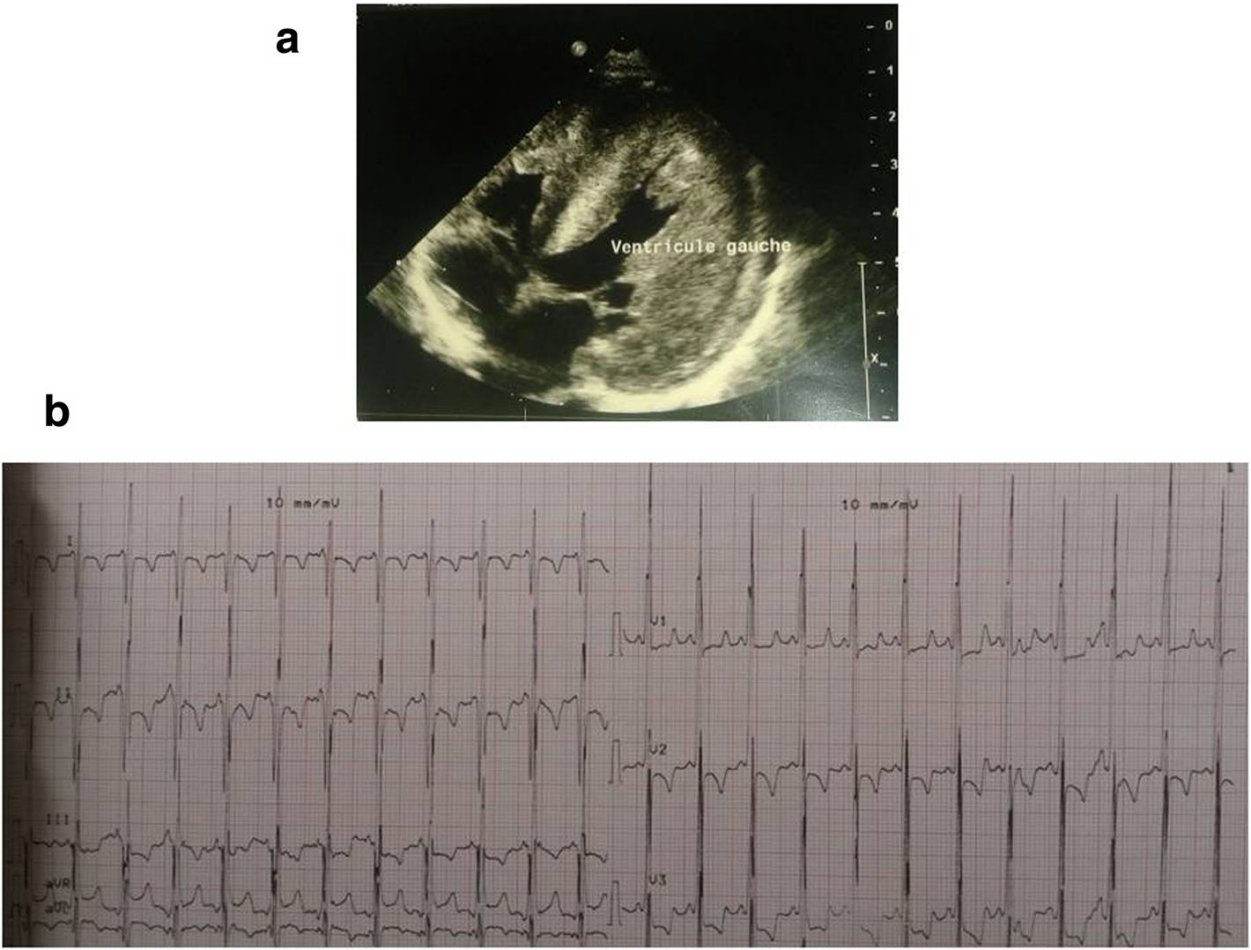
**a** Echocardiogram and **b** electrocardiography of the patient

**Fig. 2:**
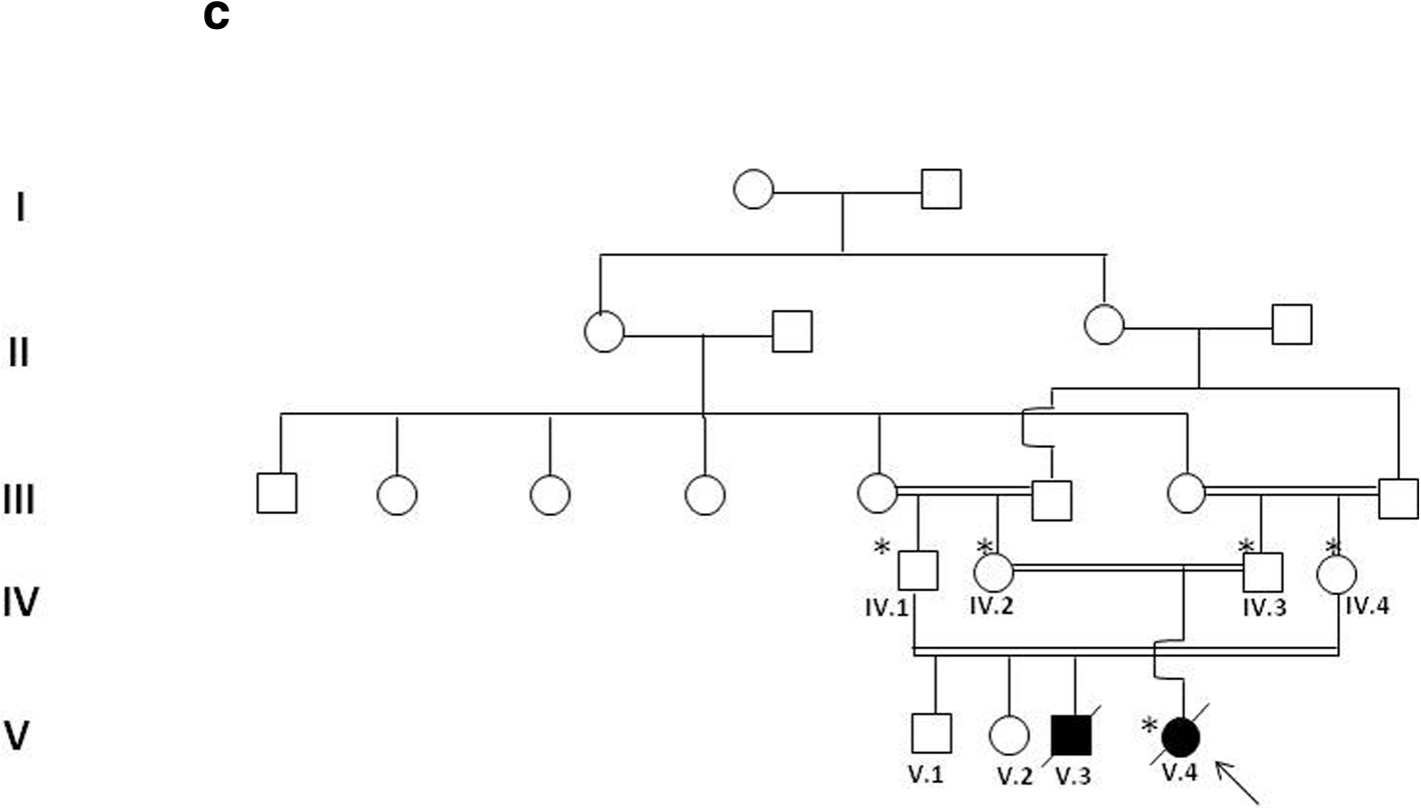
Pedigree of the studied family. The proband is indicated by an arrow

A young consanguineous Moroccan couple (IV.2 and IV.3) was referred to the department of medical genetics in Rabat because of a family history of cardiomyopathy and sudden deaths (Fig. 1). They were the parents of a baby girl (V.4) diagnosed at 7 months of life as having isolated severe concentric hypertrophy without outflow obstruction and an ejection fraction of 70% (Fig. 2). The heart was structurally normal and no valvular anomalies were observed. She had generalized hypotonia without facial dysmorphia or other associated abnormalities. She died a few days after genetic consultation without prior evaluation. In the family history, she had a double first cousin (V.3) also diagnosed as having HCM and he died suddenly at 8 months of age. No metabolic screening was performed in both patients. An ethylenediaminetetraacetic acid (EDTA) blood sample of the proband was performed with parental consent before she died. The parents of V.4 (IV.2 and IV.3) and the aunt and uncle of V.4 (IV.1 and IV.4), who the parents of V.3, provided their blood samples after consent. Because of the significant family history and the heterogeneity of pediatric cardiomyopathies, we performed WES in order to establish a potential post-mortem diagnosis in the proband.

### WES and data analysis

Genomic deoxyribonucleic acid (DNA) was extracted from peripheral blood samples following the instructions of the manufacturer (Invitrogen™; Life Technologies/Thermo Fisher Scientific). Enzymatic fragmentation was performed using KAPA HyperPlus Kit (Kapa Biosystems, Inc.). A total of 500 ng of fragmented DNA was subjected to amplification and enrichment using SeqCap EZ Human Exome v3.0 kit (Roche NimbleGen, Inc.). The 64 enriched megabases (Mb) were sequenced using an Illumina HiSeq 2500 system in rapid run paired-end mode: 2x 100 base pairs (bp). For bio-informatics analysis, bcl2fastq v1.8.4 (Illumina) was used to convert the raw data (bcl files) to fastq files. Sequences were analyzed as recommended by Genome Analysis Toolkit (GATK) best practices: mapping was performed using BWA-MEM, variant calling using GATK (haplotype caller). Annotation and filtering steps were performed using VariantStudio (Illumina). To analyze the results, the variant files of parents and index case were confronted and only variants that fulfilled recessive inheritance pattern were selected. All the variants with allele frequencies above 1% in Exome Sequencing Project (ESP) 6500 or not predicted to be deleterious were excluded.

#### Sanger sequencing

To confirm the mutation detected by WES and to perform segregation analysis, Sanger sequencing was performed. Standard polymerase chain reaction (PCR) was carried on index case’s and parents’ DNA by using the forward 5′-CGCGGTTGATGTCTCAGAGCTGC-3′ and reverse 5′-ACCCCACCCTTGTGAGGTGC-3′ primer pair in the exon 2 of *GAA* gene. PCR products were purified using ExoSAP and analyzed by standard Sanger dideoxy nucleotide sequencing using 3130 Genetic Analyzer (Thermo Fisher Scientific).

**Fig. 3:**
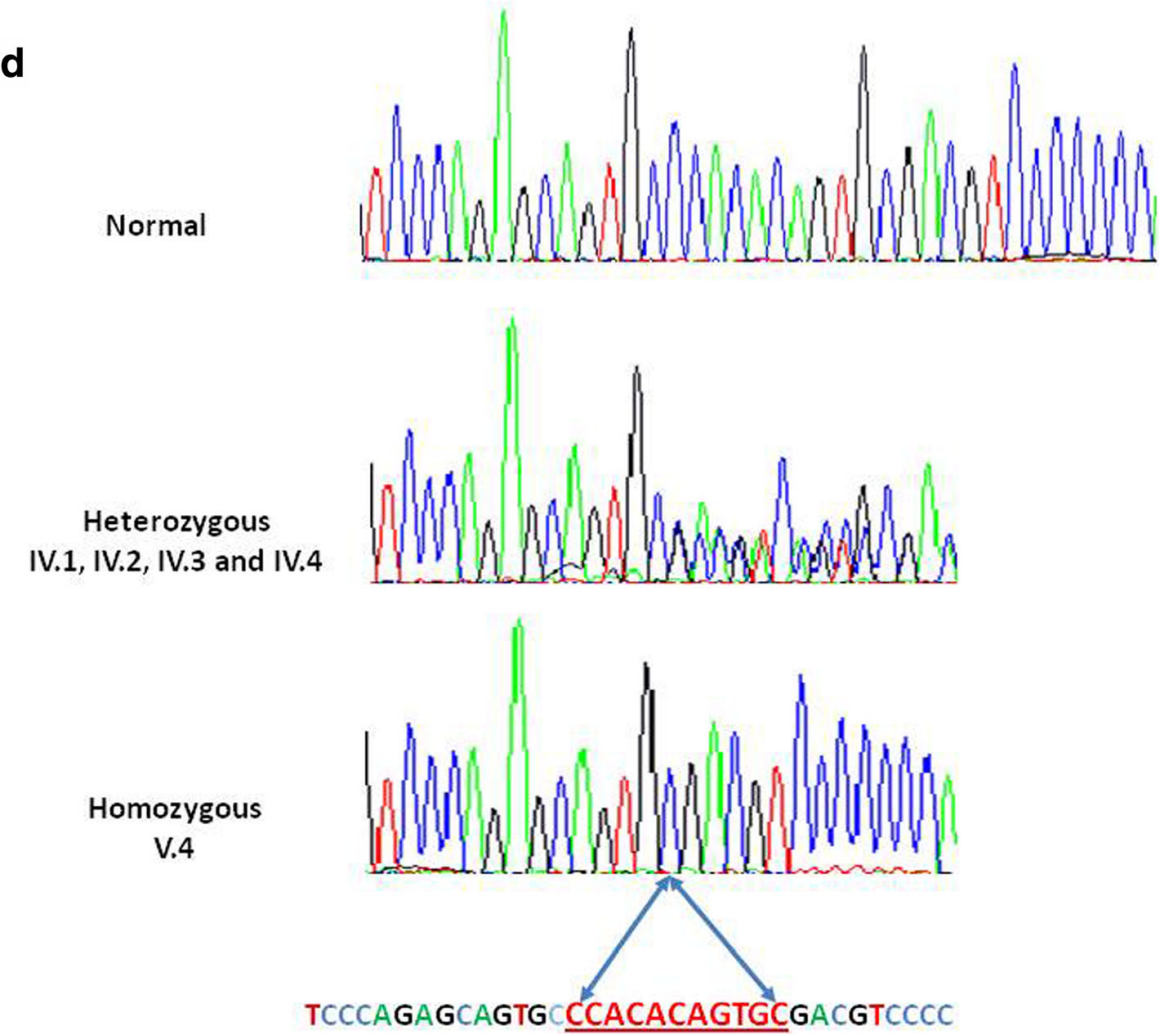
Electropherograms of the identified c.236_246del mutation. The proband (V.4) presented wih the homozygous c.236_246del mutation and both parents (IV.2 and IV.3) and her uncle and aunt (IV.1 and IV.4) are heterozygous

In our selection, WES detected only one variant in *GAA* gene (c.236_246delCCACACAGTGC; p.Pro79ArgfsX13). The *GAA* gene is located on chromosome 17q25.3 and encodes acid alpha-glucosidase (GAA). As described, biallelic mutations in *GAA* gene cause autosomal recessive GSD2 or Pompe disease (OMIM, 232300). Sanger sequencing confirmed the presence of mutation c.236_246delCCACACAGTGC; p.Pro79ArgfsX13 in *GAA* gene at heterozygous state in the parents and at homozygous state in the index case, respectively (Fig. 3).

## Discussion

The GSDs are a group of inherited metabolic disorders resulting from a defect in any one of several enzymes required for either glycogen synthesis or glycogen degradation [7]. GSD2 or Pompe disease is a monogenic autosomal recessive disorder caused by a deficiency of α-1,4-glucosidase, an enzyme required for the degradation of lysosomal glycogen [8]. The disorder was initially described by Johannes Pompe in 1932 [4]. Pompe disease is purely a neuromuscular form of GSD which does not present with metabolic abnormalities because the lysosomal enzyme defect lies outside intermediary metabolism. Instead, storage of glycogen occurs mainly in skeletal muscle and leads to loss of muscle function [7]. The clinical presentation of Pompe disease is variable with respect to the age of onset and rate of disease progression. Most of the patients die within the first year of life from cardiac and/or respiratory failure [9]. Feeding difficulties, congenital generalized hypotonia, and HCM are common in infantile Pompe disease [10].

The main symptom in the family reported here was a HCM associated with hypotonia. Congenital hypotonia is a common symptom in the neonatal period that may be environmental or genetic. Hypotonia of genetic origin might be isolated or syndromic and highly heterogeneous. In this study, spinal muscular atrophy, one of the major causes of neonatal hypotonia in Morocco, was excluded in our patient by molecular analysis of *SMN* gene [11, 12].

Pediatric hypertrophic cardiomyopathies might be isolated or associated with other abnormalities. They are due to genetic or acquired causes and are responsible for high morbidity and mortality. Their heterogeneity and the limited availability of specific metabolic and genetic tests complicate their diagnosis, thus, a specific cause can only be identified in one third of pediatric cases [13].

The advent of next-generation sequencing (NGS) technology has increased sequencing capacity and lowered the cost of sequencing, allowing for the detection of genetic causes of genetic diseases. It is a powerful alternative to Sanger sequencing for diseases with clinical variability and genetic heterogeneity like cardiomyopathies [14, 15].

In our case, we used WES for a post-mortem diagnosis in a family with HCM and sudden cardiac death and identified Pompe disease as the underlying cause. The *GAA*, c.236_246del; p.Pro79ArgfsX13 homozygous mutation identified in our patient was reported previously once in the literature by Rachel E. Palmer *et al.* in 2007 in one Spanish-Italian male patient; the patient had hypotonia at 2 months and after 6 months he was diagnosed as having cardiomyopathy, hypotonia, hepatomegaly, and macroglossia, and died at 10 months [16]. Sanger sequencing confirmation showed that our patient was homozygous and both her parents (IV.2 and IV.3) were heterozygous. Her aunt and uncle (IV.1 and IV.4), whose son died with a similar phenotype, were also found to be heterozygous for the same mutation. No sample of their affected dead child was available, but we assume that he was also affected by Pompe disease.

## Conclusion

This case illustrates the use of exome sequencing as a systematic and unbiased diagnostic tool in a pediatric case with HCM, for an appropriate management of patients and genetic counseling of their families.
